# MicroRNA‐20a in extracellular vesicles derived from duodenal fluid is a possible biomarker for pancreatic ductal adenocarcinoma

**DOI:** 10.1002/deo2.333

**Published:** 2024-03-02

**Authors:** Takashi Taniguchi, Noboru Ideno, Tomoyuki Araki, Shun Miura, Masahiro Yamamoto, Tomoki Nakafusa, Nobuhiro Higashijima, Takeo Yamamoto, Koji Tamura, So Nakamura, Toshiya Abe, Naoki Ikenaga, Kohei Nakata, Kenoki Ohuchida, Yoshinao Oda, Takao Ohtsuka, Masafumi Nakamura

**Affiliations:** ^1^ Department of Surgery and Oncology Graduate School of Medical Sciences Kyushu University Fukuoka Japan; ^2^ Department of Anatomic Pathology Graduate School of Medical Sciences Kyushu University Fukuoka Japan; ^3^ Department of Digestive Surgery Breast and Thyroid Surgery Graduate School of Medical and Dental Sciences Kagoshima University Kagoshima Japan

**Keywords:** duodenal fluid, early detection, extracellular vesicle, microRNA, pancreatic ductal adenocarcinoma

## Abstract

**Background:**

Pancreatic ductal adenocarcinoma (PDAC) has a high mortality rate owing to its late diagnosis and aggression. In addition, there are relatively few minimally invasive screening methods for the early detection of PDAC, making the identification of biomarkers for this disease a critical priority. Recent studies have reported that microRNAs in extracellular vesicles (EV‐miRs) from bodily fluids can be useful for the diagnosis of PDACs. Given this, we designed this study to evaluate the utility of cancer EVs extracted from duodenal fluid (DF) and their resident EV‐miRs as potential biomarkers for the detection of PDAC.

**Methods:**

EV‐miRs were evaluated and identified in the supernatants of various pancreatic cancer cell lines (Panc‐1, SUIT2, and MIAPaca2), human pancreatic duct epithelial cells, and the DF from patients with PDAC and healthy controls. EVs were extracted using ultracentrifugation and the relative expression of EV‐miR‐20a was quantified.

**Results:**

We collected a total of 34 DF samples (27 PDAC patients and seven controls) for evaluation and our data suggest that the relative expression levels of EV‐miR‐20a were significantly higher in patients with PDAC than in controls (*p* = 0.0025). In addition, EV‐miR‐20a expression could discriminate PDAC from control patients regardless of the location of the tumor with an area under the curve values of 0.88 and 0.88, respectively.

**Conclusions:**

We confirmed the presence of EVs in the DF and suggest that the expression of EV‐miR‐20a in these samples may act as a potential diagnostic biomarker for PDAC.

## INTRODUCTION

Although surgical resection is the only curative treatment for pancreatic ductal adenocarcinoma (PDAC), up to 85% of patients with PDAC are diagnosed with unresectable disease, resulting in a 5‐year survival rate of less than 10%.^1^, ^2^ However, previous studies have reported favorable prognosis in patients with incidentally detected non‐symptomatic PDAC, particularly in those with lesions of less than one centimeter.^3^, ^4^, ^5^ This suggests that the development of biomarkers for the early detection of PDAC should be treated as a priority to help improve prognosis in these patients.

Pancreatic juice cytology during endoscopic retrograde pancreatography (ERP‐PJC) and endoscopic ultrasound‐guided fine‐needle aspiration (EUS‐FNA) are both often used to confirm the presence of PDAC. Because of its high diagnostic accuracy, EUS‐FNA has become the gold standard for pathological confirmation of PDAC,^6^, ^7^ whereas routine endoscopic retrograde cholangiopancreatography is not normally recommended because of the higher occurrence of post‐ERP pancreatitis (PEP) and lower sensitivity of pancreatic juice (PJ) cytology.^8^, ^9^ Despite this, PJ is still a significant source of PDAC‐associated biomarkers as it contains DNA, RNA, proteins, and EVs derived from neoplastic cells and has the potential to increase the sensitivity/specificity of biomarker evaluations when compared to other bodily fluids, such as blood, urine, and saliva.^10^ The number of studies focused on duodenal fluid (DF) with or without secretin stimulation for DNA/protein marker tests has been increasing as DF usually contains sufficient PJ to allow for biomarker evaluation, and DF collection is significantly easier and less invasive than endoscopic retrograde cholangiopancreatography and EUS‐FNA.^11^, ^12^, ^13^ Our previous study demonstrated that enough DF can be collected without secretin stimulation and can facilitate S100 calcium‐binding protein measurement and the discrimination of PDAC samples when evaluated using a satisfactory diagnostic screening test.^14^ Thus, we hypothesized that molecules within the EVs secreted into PDAC PJ could be significant biomarkers for this disease as they often include specific biomarkers. One example of this is a surface proteoglycan, glypican‐1, which is enriched on cancer cell‐derived EVs from the blood and can be used to distinguish healthy subjects from patients with early‐stage PDAC.^15^ In addition, our previous study showed that the extracellular vesicle‐derived microRNAs (miRNAs; EV‐miRs) EV‐miR‐21, and EV‐miR‐155 are useful markers for discriminating patients with chronic pancreatitis (CP) from those with PDAC.^16^ Thus, the aim of this study was to purify cancer EVs from DF and to identify any relevant biomarkers for the diagnosis of PDAC via the close examination of DF‐derived EV‐miRs.

## METHODS

### EV‐miR collection from cultured cell supernatant

We collected the serum‐free medium supernatants of three pancreatic cancer cell lines, Panc‐1, MIAPaca2, and SUIT2, and human pancreatic duct epithelial cells (HPDE) and centrifuged at 2000×*g* for 20 min at 4°C to remove cells and filtered through a 200 nm filter. This filtrate was ultracentrifuged at 170,000×*g* for 70 min at 4°C and the precipitate was collected as EVs. This precipitate was resuspended in 40 µL of phosphate‐buffered saline (PBS) and then used in downstream evaluations.^17^, ^18^

miRNAs were extracted from these purified EVs using the miRNeasy Mini Kit (Qiagen) according to the manufacturer's instructions. The RNA volume was normalized to 40 µL and the RNAs were quantified using a NanoDrop ND‐1000 spectrophotometer (NanoDrop Technologies).

### Patients and DF collection

This study was approved by the Ethics Committee at Kyushu University (no. 30–230) and conducted according to the Ethical Guidelines for Human Genome/Gene Research enacted by the Japanese Government and the Helsinki Declaration. Written informed consent was obtained from each patient.

Thirty‐four consecutive subjects, including 27 patients with PDAC and seven patients with neither pancreatic disease nor malignancy, were enrolled in the study. DF samples were collected within 3 min during endoscopic retrograde cholangiopancreatography or gastrointestinal endoscopy at Kyushu University Hospital from July 2013 to February 2015. The diagnosis was confirmed by pathological or cytological assessment and a cOmplete ULTRA Protease Inhibitor Tablet (Roche) was added to the samples and immediately transferred to 2 mL tubes before being stored at −80°C until assay, as previously reported.^14^

### Isolation of DF EVs

Note that, 500 µL of DF was centrifuged at 300×*g* for 10 min at 4°C, and the supernatant was centrifuged at 16,500×*g* for 20 min at 4°C. Next, the supernatant was filtered through a 200 nm filter and the filtrate was ultracentrifuged at 170,000×*g* for 70 min at 4°C. The precipitate was collected as EVs and resuspended in 40 µL of PBS. EVs were observed using transmission electron microscopy, and nanoparticle size distribution and concentration were evaluated using nanoparticle tracking analysis (NTA) on a NanoSight LM10‐HS instrument (Nanosight). We then confirmed the designation of these particles as EVs via the evaluation of CD‐81, which is normally expressed in EVs,^17^ on these particles using a western blot and an anti‐CD81 antibody. miRNAs were extracted from these purified EVs as previously described.^16^

### Evaluating EV‐miR expression

Quantitative reverse transcription polymerase chain reaction (qRT‐PCR) was performed in accordance with the manufacturer's instructions. We evaluated miR‐16 and miR‐20a expression using two‐step qRT‐PCR and primers specific for miR‐16, miR‐20a, miR‐21, miR‐155, and miR‐191. PCR amplifications were performed in triplicate and we used the ΔCT method to calculate the relative miRNA expression in each sample.

### Evaluating miR‐20a expression in tissue samples

We randomly selected specimens of PDAC (*n* = 5), intraductal papillary mucinous neoplasm (IPMN)‐low grade (IPMN‐LG, *n* = 5), CP (*n* = 5), and normal pancreas (NP, *n* = 5), which were selected this study period. miRNA was extracted using the macro‐dissection technique as we have previously described.^19^, ^20^ We evaluated miR‐16 and miR‐20a expression as described above.

### Statistical analyses

Statistical analyses were performed using JMP Pro 15 (SAS Institute, Inc.) and the Wilcoxon signed‐rank test was used to compare continuous and categorical variables. Receiver operating characteristic (ROC) curves were generated, and the area under the curve (AUC), sensitivity, and specificity values were calculated to evaluate the diagnostic value of candidate miRNAs. The cutoff point was determined using the Youden index and differences between groups were considered significant when the bilateral *p*‐value was less than 0.05.

## RESULTS

### Confirming the presence of EVs in DF samples

**FIGURE 1:**
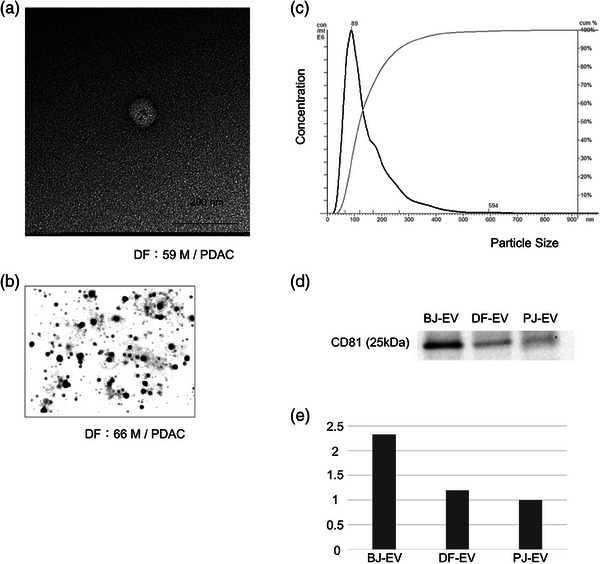
Confirmation of the presence of extracellular vesicles (EVs) in duodenal fluid samples. (a) Transmission electron microscopy revealed small vesicles in the duodenal fluid from various samples when evaluated using the negative stain method. (b) Small vesicles were visualized using nanoparticle tracking analysis. (c) The mode of the particle size distribution was 89 nm, and their concentration was 3.43×10^11^ particles/mL. (d) EVs isolated from bile juice, duodenal fluid, and pancreatic juice were analyzed by western blotting using anti‐CD81 antibodies, which confirmed their designation as EVs. (e) CD81 expression levels of EVs isolated from bile juice, duodenal fluid, and pancreatic juice were semi‐quantified.

Transmission electron microscopy analysis revealed 50–100 nm rounded vesicles, consistent with the features of EVs (Figure 1a). Subsequent NTA revealed abundant small vesicles (Figure 1b) with sizes normally distributed around 89 nm and an overall concentration of 3.43×10^11^ particles/mL (Figure 1c). Western blotting demonstrated the expression of CD81 (Figure 1d), but no expression of calnexin and GM130 at either speed of ultracentrifugation (Figure S1), which confirmed the presence of EVs and the absence of other intracellular components.^21^ We further semi‐quantified the CD81 expression level of EVs derived from DF, bile juice, and PJ (Figure 1e), and hypothesized that about 30% of EVs in DFs were derived from PJ.

### Identifying an internal control for the analyses of EV‐miR expression in DF samples

**FIGURE 2:**
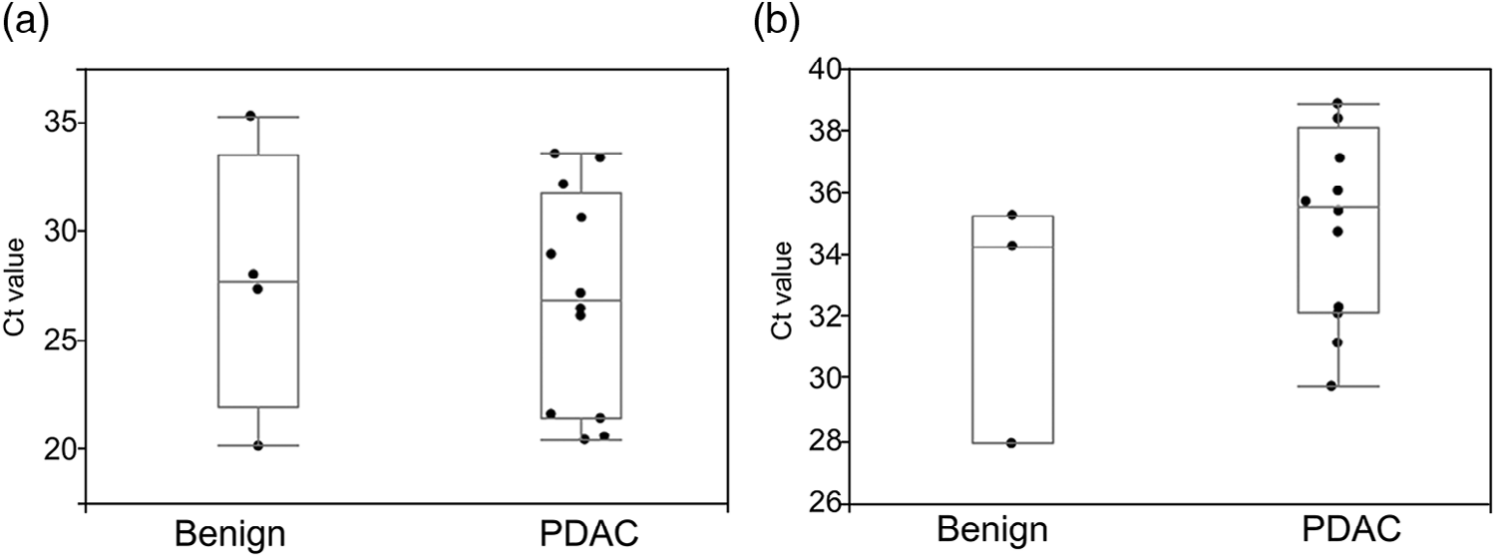
Identification of internal control for the analyses of EV‐miR expression in duodenal fluid samples. (a) Comparison of EV‐miR‐16 expression in four benign disease and 12 PDAC patients using threshold cycle (Ct) values. No significant difference was observed (*p* = 0.86, median Ct = 27.1 [20.0–35.2], Wilcoxon signed‐rank test was used). (b) Comparison of EV‐miR‐191 expression in four benign disease and 12 PDAC patients using Ct values. No significant difference in expression despite the fact that EV‐miR‐191 was not detected in one of the benign samples (*p* = 0.22, median Ct = 35.2 [28.0–38.8]). EV‐miR, microRNAs in extracellular vesicles; PDAC, pancreatic ductal adenocarcinoma.

Because internal controls for EV‐miR expression analyses in DF have not been reported, we tested EV‐miR‐191 and EV‐miR‐16 as internal controls based on previous findings.^16^, ^20^ This was completed by comparing the expression levels of EV‐miR‐191 and EV‐miR‐16 in DF from 4 benign disease and 12 PDAC patients using threshold cycle (Ct) values. There were no significant differences in the Ct values of either EV‐miR‐191 (*p =* 0.22) or EV‐miR‐16 (*p =* 0.86) among these samples but EV‐miR‐191 was not detected in one benign sample, while EV‐miR‐16 was detected in all samples at significantly lower Ct values than those of EV‐miR‐191 (EV‐miR‐191, median Ct = 35.2 [28.0–38.8]; EV‐miR‐16, median Ct = 27.1 [20.0–35.2]; *p* < 0.001; Figure 2). Therefore, EV‐miR‐16 was selected as the internal control for the remainder of this study.

### Evaluating EV‐miR expression in cultured cell supernatants and biomarker selection

**FIGURE 3:**
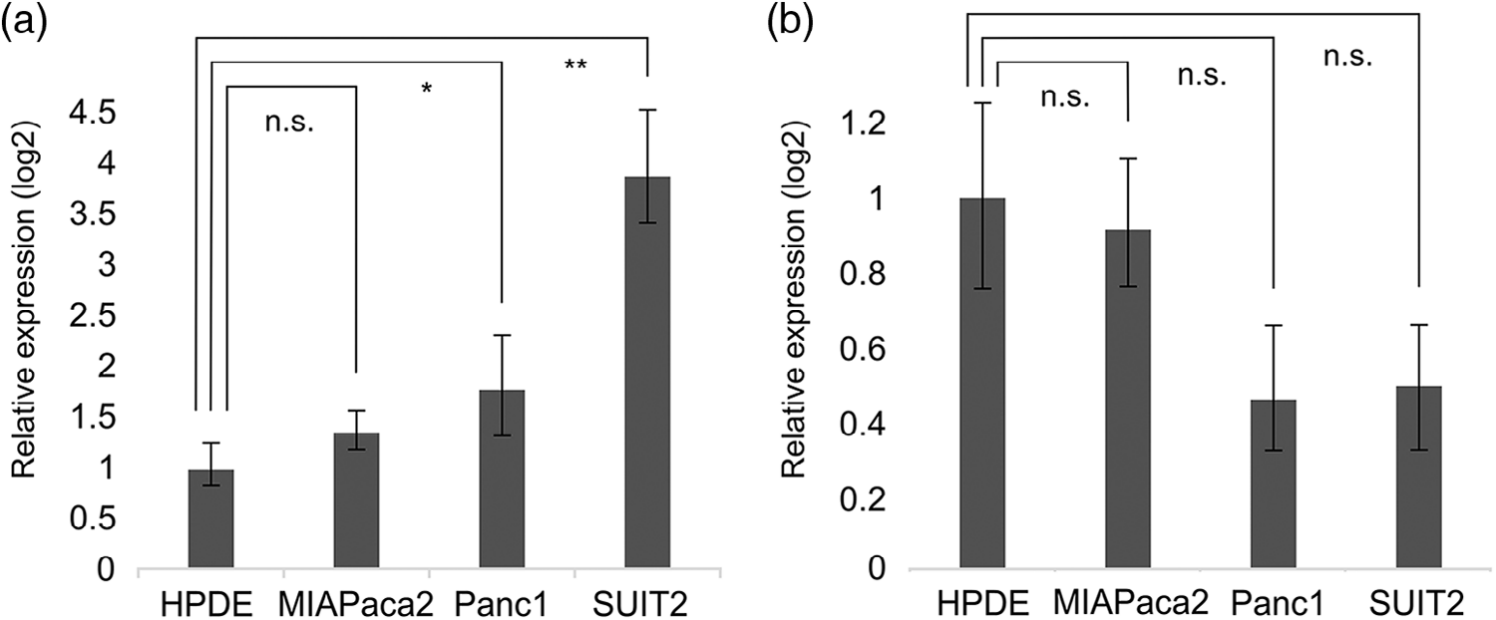
Comparison of microRNAs in extracellular vesicles (EV‐miR)‐20a expression in various cancer cell lines. (a) Comparisons of the expression of EV‐miR‐20a in human pancreatic duct epithelial cells (HPDE), MIAPaca2, Panc1, and SUIT2 revealed an increase in EV‐miR‐20a in MIAPaca2, Panc1, and SUIT2 when compared to HPDE samples. *p = 0.03, **p = 0.006, Wilcoxon signed‐rank test was used. n.s., not significant. (B) Expression of EV‐miR‐21 in HPDE, Panc1, MIAPaca2, and SUIT2. No significant differences were observed between these groups.

Given the data described in previous reports, we continued our evaluations using EV‐miR‐21, EV‐miR‐155, and EV‐miR‐20a as potential diagnostic markers for PDAC and examined whether they were present in the EVs secreted from pancreatic cancer cells. Although EV‐miR‐155 was not detected in cell supernatants, both EV‐miR‐21 and EV‐miR‐20a were expressed in all cell supernatants. A comparison of this data then revealed that EV‐miR‐20a expression was significantly upregulated in two of the three pancreatic cancer cell lines when compared to the HPDE control (Figure 3). We then went on to select EV‐miR‐20a as our target for our DF experiments.

### EV‐miR‐20a expression in DF samples

**FIGURE 4:**
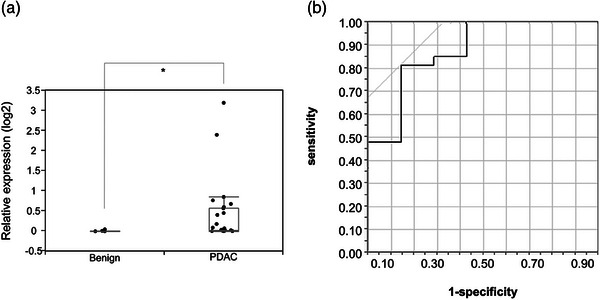
Comparison of microRNAs in extracellular vesicles (EV‐miR)‐20a expression in 22 pancreatic ductal adenocarcinoma (PDAC) and seven control samples. (a) Relative expression levels of EV‐miR‐20a in the duodenal fluid were significantly higher in PDAC patients than in the control. *p = 0.006, Wilcoxon signed‐rank test was used. (b) ROC curve for EV‐miR‐20a expression in PDAC samples producing an AUC value of 0.85. ROC, receiver operating characteristic; AUC, area under the curve.

Sufficient DF EVs for EV‐miR analysis were available in all 34 subjects. These evaluations revealed that EV‐miR‐20a was significantly upregulated in patients with PDAC when compared to the controls (*p* = 0.0025; Figure 4a) and that the AUC value for EV‐miR‐20a was 0.88 (Figure 4b). We then used the Youden index to determine the cut‐off value for our ROC assays and we were able to stratify our patients into two groups: positive and negative (Table 1). Additionally, although ERP‐PJC failed to produce sufficient PJ from two patients, DF samples were available for all 34 patients and revealed a sensitivity of 82% (95% confidence interval [CI]; 63–92%) and a specificity of 86% (95% CI; 49–97%) for PDAC compared to only 70% sensitivity using ERP‐PJC.

**TABLE 1:** Clinical details of the 29 patients in our cohort, of all Asian races, including 22 pancreatic ductal adenocarcinoma patients and seven controls.

	UICC Stage disease	Location	CA19‐9 (U/mL)	EV‐mir‐20a	ERP‐PJC
PDAC‐1	IIB	Pt	365.3	Positive	Negative
PDAC‐2	IB	Pb	19.3	Positive	Positive
PDAC‐3	IIB	Pb	2221	Positive	Positive
PDAC‐4	IIB	Pb	33.3	Positive	Positive
PDAC‐5	IIB	Ph	11.2	Positive	Positive
PDAC‐6	III	Ph	543.5	Positive	Positive
PDAC‐7	IV	Pb	10.0	Positive	Positive
PDAC‐8	IIA	Ph	184.6	Negative	Positive
PDAC‐9	IIB	Ph	117.8	Positive	Negative
PDAC‐10	IA	Ph	76.8	Positive	Positive
PDAC‐11	IIB	Ph	25464.0	Positive	Positive
PDAC‐12	IIB	Ph	787.9	Positive	Positive
PDAC‐13	IIB	Ph	937.6	Positive	n.a.
PDAC‐14	IIB	Ph	19.1	Negative	Positive
PDAC‐15	IV	Ph	285.9	Positive	Positive
PDAC‐16	III	Ph	75.1	Positive	n.a.
PDAC‐17	III	Ph	233.1	Positive	Negative
PDAC‐18	IV	Pb	162.3	Negative	Positive
PDAC‐19	IV	Pb	401.0	Positive	Negative
PDAC‐20	IB	Ph	154.2	Negative	Positive
PDAC‐21	IV	Ph	101.6	Positive	Positive
PDAC‐22	IIA	Ph	93.9	Negative	Negative
PDAC‐23	IIB	Pb	1578	Positive	Positive
PDAC‐24	IIB	Ph	35.7	Positive	Negative
PDAC‐25	IIA	Ph	269	Positive	Positive
PDAC‐26	IIA	Ph	2.2	Positive	Positive
PDAC‐27	IIA	Ph	561	Positive	Positive
Benign‐1	Benign	‐	7.3	Negative	n.a.
Benign‐2	GB polyp	‐	14.2	Positive	n.a.
Benign‐3	GB polyp	‐	0.6	Negative	n.a.
Benign‐4	CBD stones	‐	13.8	Negative	n.a.
Benign‐5	CBD stones	‐	5.5	Negative	n.a.
Benign‐6	Donor	‐	5.3	Negative	n.a.
Benign‐7	Donor	‐	8.4	Negative	n.a.

Abbreviations: CA19‐9, carbohydrate antigen 19‐9 (<37.0 U/mL); CBD, common bile duct; ERP‐PJC, pancreatic juice cytology during endoscopic retrograde pancreatography; EV‐miR, extracellular vesicle‐microRNA; GB, gall bladder; n.a., not available; Pb, pancreatic body; PDAC, pancreatic ductal adenocarcinoma; Ph, pancreatic head; Pt, pancreatic tail; UICC, Union for International Cancer Control.

**FIGURE 5:**
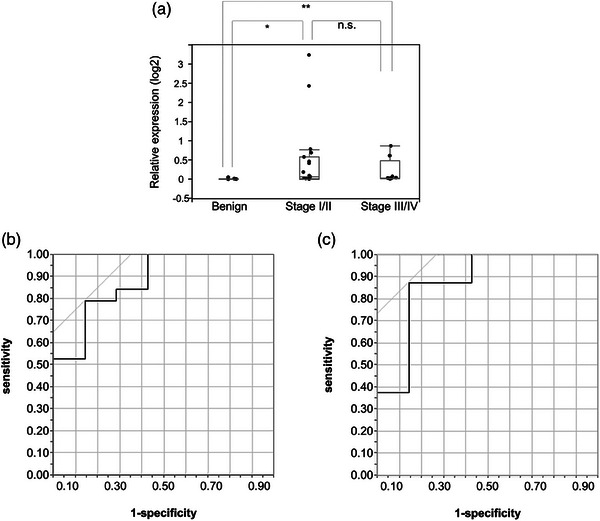
Comparisons of microRNAs in extracellular vesicles (EV‐miR)‐20a expression in patients with early‐ (stage I/II) or advanced‐stage (stage III/IV) PDAC and healthy controls. (a) Relative expression levels of EV‐miR‐20a in early‐ and advanced‐stage PDAC were significantly higher than those in the control patients (14 of early‐stage, eight of advanced‐stage PDAC, and seven of controls were included). *p = 0.015, **p = 0.018, Wilcoxon signed‐rank test was used. (b) ROC curve for EV‐miR‐20a expression in early‐stage PDAC producing an AUC value of 0.84. (c) ROC curve for EV‐miR‐20a expression in advanced‐stage PDAC producing an AUC value of 0.88. ROC, receiver operating characteristic; AUC, area under the curve; PDAC, pancreatic ductal adenocarcinoma.

This suggested that this transcript may serve some diagnostic purpose and therefore we compared the expression levels of EV‐miR‐20a in the following three groups: early‐stage (stages I and II), advanced‐stage (stages III and IV), and control patients. These experiments showed that the expression of EV‐miR‐20a was significantly higher in early‐ and advanced‐stage patients when compared to the controls (early‐stage patients, *p =* 0.004; advanced‐stage patients, *p =* 0.018), but this difference was not significant between early‐ and advanced‐stage patients (*p =* 0.98; Figure 5a). Additional ROC curve analysis revealed that EV‐miR‐20a also demonstrated a high degree of diagnostic accuracy for PDAC patient groups (early‐stage patients, AUC = 0.88; advanced‐stage patients, AUC = 0.88), confirming its general utility as a diagnostic biomarker for this disease (Figure 5b,c).

**FIGURE 6:**
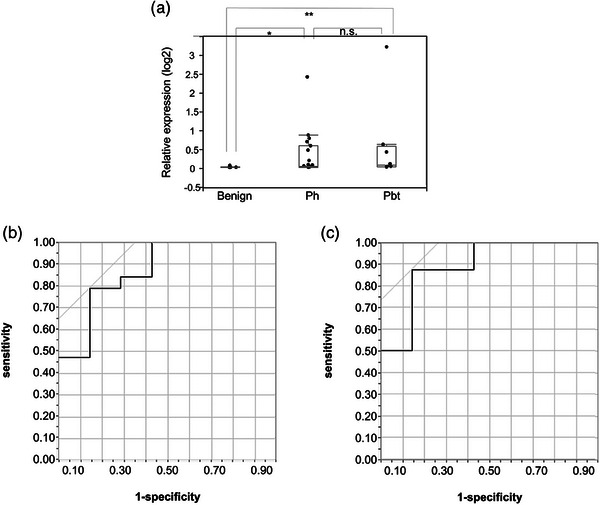
Expression of microRNAs in extracellular vesicles (EV‐miR)‐20a in patients with PDAC centered at various locations including the pancreas head (Ph) and pancreas body and tail (Pbt). (a) Relative expression levels of EV‐miR‐20a from Ph and Pbt samples were significantly higher than those from control patients (15 Ph and seven Pbt samples were used). *p = 0.014, **p = 0.021, Wilcoxon signed‐rank test was used. (b) ROC curve for EV‐miR‐20a expression in Ph samples producing an AUC value of 0.84. (c) ROC curve for EV‐miR‐20a in Pbt samples producing an AUC value of 0.88. ROC, receiver operating characteristic; AUC, area under the curve.

We then went on to evaluate the differences in EV‐miR‐20a expression based on the location of the tumor, with these being divided into two groups: pancreas head (Ph) or pancreas body and tail (Pbt) tumors. Despite this stratification, we saw no significant differences in the expression of this biomarker between Ph and Pbt samples (*p* = 0.61; Figure 6a). In addition, the ROC analysis of this data revealed that EV‐miR‐20a showed high accuracy for both Ph and Pbt samples (Ph: AUC = 0.87; Pbt: AUC = 0.89; Figure 6b,c).

### MiR‐20a expression in tissue samples

MiR‐20a from tissue samples, including NP, PDAC, CP, and IPMN‐LG, were analyzed and the expression levels in PDAC were not significantly higher than those in NP, CP, and IPMN‐LG, respectively. MiR‐20a expression levels from PDAC, IPMN‐LG, and CP were higher than those from NP, although a significant difference was shown only between IPMN‐LG and NP (Figure S2).

## DISCUSSION

This study demonstrated that EV‐miRs in the DF are likely to be useful in the detection of PDAC, with EV‐miR‐20a identified as a potential biomarker for the diagnosis of this condition regardless of disease stage or tumor location.

EVs are generally described as 40–150 nm lipid bilayer membrane‐bound particles derived from specific biogenesis pathways within cells that are accessible within the plasma of circulating peripheral blood samples.^22^ Ultracentrifugation is currently the base technique used for EV isolation; however, no previous study has reported the isolation of EVs from DF for PDAC detection.^23^ Our preliminary study revealed that a sufficient quantity of DF EVs could not be purified using the method previously described for EV‐miR profiling in pure PJ.^16^ This is probably because DF contains many types of substances other than PJ, but this issue was resolved by increasing the centrifugal force from 140,000 to 170,000×*g*, which resulted in the purification of DF EVs at high enough concentrations to facilitate further analyses.

MiRNAs have recently drawn attention as novel biomarkers for various malignant diseases, including PDAC. Although miRNAs, such as miR‐21 and miR‐155, are useful diagnostic markers of PDAC, these are all extracted from cell pellets and require secretin induction to produce sufficient (10–20 mL) PJ containing enough neoplastic cells to evaluate.^20^ However, recent evidence suggests that cancer EVs and EV‐miRs can be detected even in a patient's plasma,^15^, ^24^ suggesting that it may be possible to analyze cancer EVs using extremely small amounts of PJ. Nakamura et al.^16^ successfully extracted EVs from 500 µL of PJ, and expression of EV‐miR‐21 and EV‐miR‐155 were shown to be excellent biomarkers for distinguishing PDAC from CP. The accuracies of EV‐miR‐21 and EV‐miR‐155 for PDAC diagnosis were 83% and 89%, respectively.^24^ Despite the fact that the fraction of PJ within DF samples is very small, this study showed that EV‐miR‐20a from 500 µL of DF produced high sensitivity (82%), specificity (86%), and accuracy (82%) for PDAC detection, making it comparable to EV‐miR‐21 and EV‐miR‐155 from PJ.

MiR‐20a, an oncomiRNA, is encoded within the miR‐17‐92 cluster and is closely related to cell proliferation and cancer progression.^25^ Motoyama et al.^26^ reported that tissue‐derived miR‐20a was up‐regulated in 77% (52 of 67) of colorectal cancer, while Yan et al.^27^ reported that overexpression of miR‐20a may act as a potential therapeutic target associated with the proliferation and invasion of PDAC. Lu et al.^28^ reported that higher expression levels of miR‐20a‐5p in plasma were significantly associated with gemcitabine resistance in PDAC patients. Despite of that EV‐miR‐20a was higher in early‐stage PDACs than in advanced stages. EV‐miR‐20a in DF secreted from PDAC cells might be higher in advanced‐stage PDACs compared to early‐stages, considering that miR‐20a may be related to malignant behavior of PDACs.^27^, ^28^ However, main pancreatic duct obstruction and pancreatic exocrine insufficiency would be significantly associated with advanced PDACs, which could reduce the proportion of PJ in DF. Therefore, the DF test might have an advantage in the detection of early‐stage PDACs.

We also analyzed miR‐20a in tissue samples to evaluate the usefulness of miR‐20a to discriminate PDACs from other high‐risk individuals. MiR‐20a in PDAC, IPMN‐LG, and CP were higher than those in NP, although a significant difference was shown only between IPMN‐LG and NP. We interpreted these data as follows. One is that miR‐20a is a potential biomarker to discriminate not only PDAC patients but also high‐risk individuals from those without pancreatic disease. Another is that there might be a discrepancy between the expression of miR20a in EVs and that in tissue samples because we extracted miRNA from the formalin‐fixed, paraffin‐embedded sample using macro‐dissection, which contains various types of cells except for neoplastic cells. We did not perform lazar micro‐dissection to selectively obtain cancer cells because RNA quality using this procedure would be significantly low and unable to undergo downstream analyses such as qPCR. Therefore, confirmation of EV‐miRs released from cultured cells seems to be a significant finding. Either way, a validation study would be required to investigate that miR20 may be a marker for the presence of some pancreatic disease, including benign, rather than a marker for pancreatic cancer.

A recent study investigating DF collected during EUS with secretin stimulation demonstrated that EV‐miR‐21, 25, and 16 were useful for PDAC detection.^29^ This study also selected miR‐155 as a PDAC biomarker candidate because the previous study showed higher levels of miR‐155 in PJ and EV from PDAC patients’ PJ/serum compared to those in controls.^16^, ^30^, ^31^ In this study cohort, EV‐miR‐155 in DF of PDAC were not significantly higher than those of control, which was inconsistent with our previous report.^16^ These findings indicated the requirement of a multi‐center, international validation study among various racial/ethnic groups as we have done in our previous study.^14^

Despite its clear value, there are several limitations to this study. It was a retrospective study with a small sample size. Validation with larger study populations is needed to determine the cut‐off value for EV‐miR‐20a expression in DF in the discrimination of PDAC patients from controls in the setting of a prospective study. The expression levels of EV‐miR‐20a in pure PJ were not examined because of a lack of matched pure PJ/DF samples and our study protocol did not allow cannulation of the main pancreatic duct in participants without pancreatic disease. Internal controls for miRNA expression analyses are uncertain, we selected miR‐16 out of two candidates.^16^, ^20^ The Presence of the other biological fluid and the difference in the number of tumor‐released EVs would result in the variation of CT value of miR‐16 among study cohorts.

Due to the sample availability, DF samples were collected and immediately froze 6 to 4 years ago; however, EVs are able to prevent RNase from degrading EV‐miRs even in feces as previously demonstrated.^32^ Furthermore, another study revealed that EV‐miR‐30c was protected from degradation in serum samples frozen for up to 14 years.^33^ Therefore, degradation by RNase and long‐term stored influence could be ignored in this study.

In conclusion, expression analyses of EV‐miR‐20a effectively discriminate between patients with PDAC and those without pancreatic disease. EVs from DF seem to contain sufficient quantities of valuable biomarkers to facilitate the development of minimally invasive PDAC screening assays, since the collection of DF is significantly less invasive than direct PJ aspiration, and its diagnostic capacity was satisfactory when the appropriate biomarker was selected. In our future studies, this biomarker will be validated with larger study populations including high‐risk individuals such as IPMN and CP.

## CONFLICT OF INTEREST STATEMENT

None.

## Supporting information


**Figure S1** Extracellular vesicles (EVs), derived from duodenal fluid (DF), and cell lysate were analyzed by western blotting. EVs, derived from DF, and cell lysate were analyzed by western blotting using anti‐CD81, ‐Calnexin, and ‐GM130 antibodies. EVs were extracted by ultracentrifugation at *140,000 and **170,000×*g*.

**Figure S2** Comparisons of miR‐20a expression in tissue samples. Comparisons of miR‐20a expression in tissue samples, including normal pancreas from specimens containing pancreatic neuroendocrine neoplasms, pancreatic ductal adenocarcinoma, chronic pancreatitis (CP), and low‐grade intraductal papillary mucinous neoplasm (IPMN). *p = 0.037, Wilcoxon signed‐rank test was used. *n.s* not significant.

Supplementary information
