# Supplementary figures and images for: MicroRNA‐20a in extracellular vesicles derived from duodenal fluid is a possible biomarker for pancreatic ductal adenocarcinoma
Source: DEN Open. 2024 Mar 2;4(1):e333. doi: 10.1002/deo2.333 (PMC10908371; doi:10.1002/deo2.333)

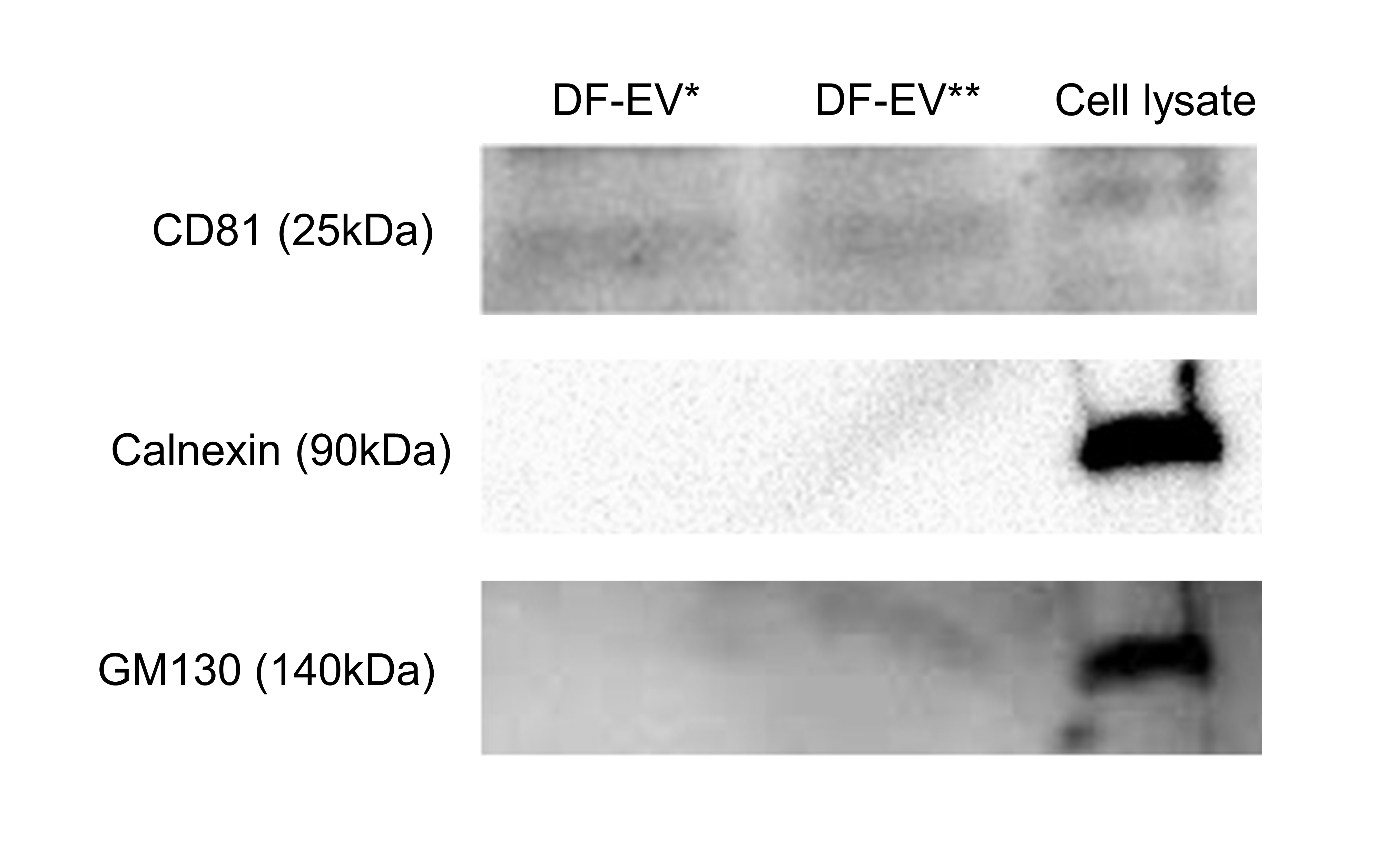

Supplement: Supplementary file 1 — Figure S1 Extracellular vesicles (EVs), derived from duodenal fluid (DF), and cell lysate were analyzed by western blotting. EVs, derived from DF, and cell lysate were analyzed by western blotting using anti‐CD81, ‐Calnexin, and ‐GM130 antibodies. EVs were extracted by ultracentrifugation at *140,000 and **170,000×g. [file DEO2-4-e333-s002.tif]

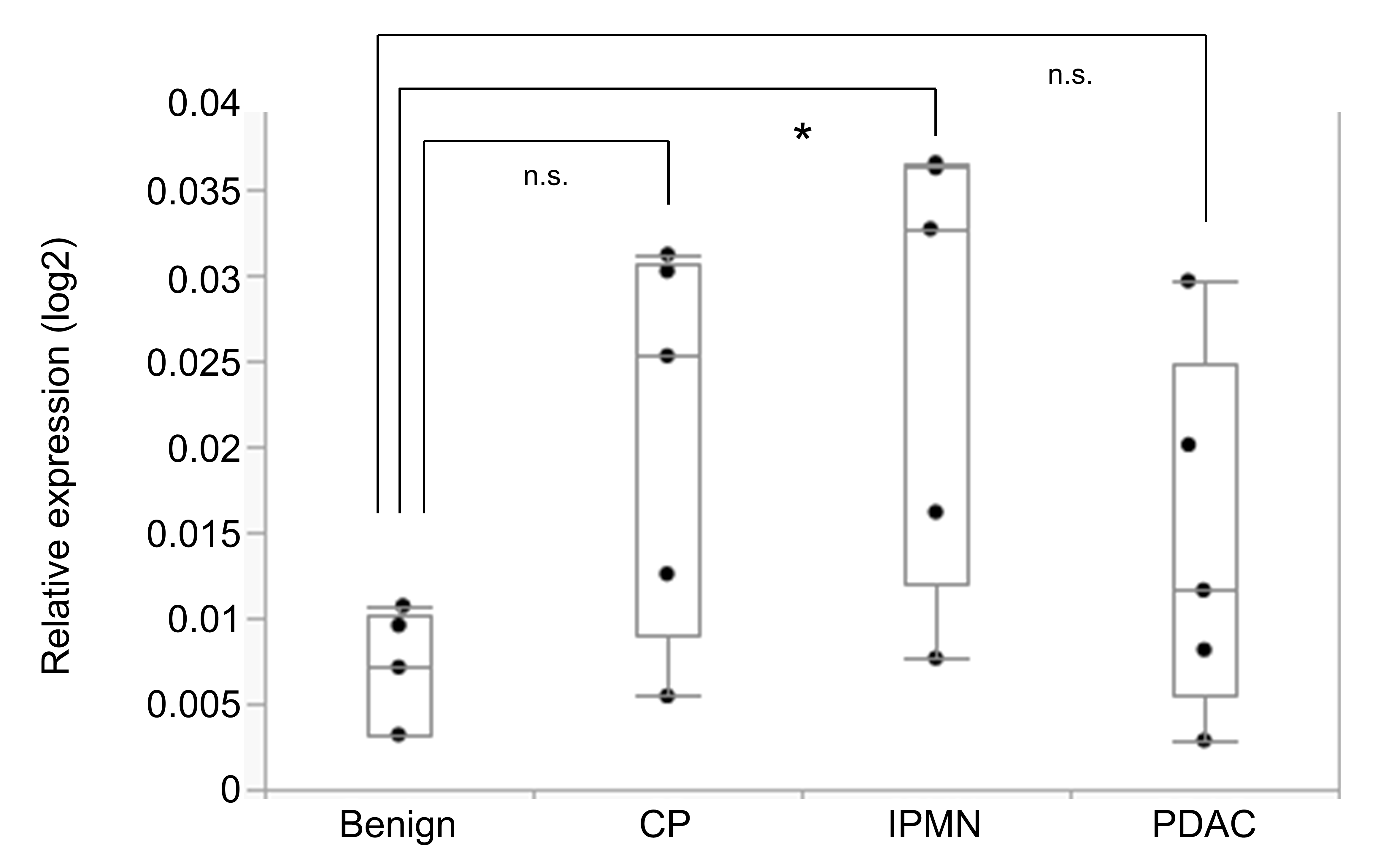

Supplement: Supplementary file 2 — Figure S2 Comparisons of miR‐20a expression in tissue samples. Comparisons of miR‐20a expression in tissue samples, including normal pancreas from specimens containing pancreatic neuroendocrine neoplasms, pancreatic ductal adenocarcinoma, chronic pancreatitis (CP), and low‐grade intraductal papillary mucinous neoplasm (IPMN). *p = 0.037, Wilcoxon signed‐rank test was used. n.s not significant. [file DEO2-4-e333-s003.tif]
