# Supplementary material for: MicroRNA‐20a in extracellular vesicles derived from duodenal fluid is a possible biomarker for pancreatic ductal adenocarcinoma
Source: DEN Open. 2024 Mar 2;4(1):e333. doi: 10.1002/deo2.333 (PMC10908371; doi:10.1002/deo2.333)
Supplement: Supplementary file 3 — Supplementary information [file DEO2-4-e333-s001.docx]

**Supplementary information**

**Methods**

**EV-miR collection from cultured cell supernatant**

Three pancreatic cancer cell lines, Panc-1 and MIAPaca2/SUIT2, were obtained from the RIKEN Bio Resource Center (Ibaraki, Japan) and Japanese Health Science Research Resources Bank (JCRB) (Osaka, Japan), respectively. Human pancreatic duct epithelial cells (HPDE) were obtained from the University of Toronto (Toronto, Canada).

Three pancreatic cancer cell lines, Panc-1, MIAPaca2, and SUIT2, were maintained in DMEM supplemented with 10% fetal bovine serum, while Human pancreatic duct epithelial cells (HPDE) were grown in HuMedia-KG2 (Kurabo Industries, Osaka, Japan) at 37℃, 5% CO_2_ in a humidified incubator. When cell confluency reached around 80%, the medium was exchanged for serum-free medium and the cells were cultured for an additional 48 h before the culture supernatants were collected, centrifuged at 2000 × *g* for 20 min at 4°C to remove cells, and filtered through a 200 nm filter. This filtrate was further ultracentrifuged at 170,000 × *g* for 70 min at 4°C and the precipitate was collected as EVs.

**Patients and DF collection**

According to the study protocol, an endoscope was inserted into the duodenum, and DF was collected within 3 minutes near the duodenal papilla without secretin stimulation while gently pushing the duodenal mucosa using an ERCP cannula (PR-130Q; Olympus Medical Systems Corp) at the beginning of procedure.

**Isolation of DF EVs**

EVs were extracted from PJ as previously described [16], and this method was modified for EV extraction from DF. Ultracentrifugation was modified 140,000g for PJ to 170,000g for DF. Anti-CD-81 antibody used for western blot was obtained from Life Technologies Corporation, Carlsbad, CA, USA; diluted 1:250.

CD81 is one of the EV markers and calnexin and GM130 are markers of endoplasmic reticulum and Goldi apparatus.

**Evaluating EV-miR expression**

Quantitative reverse transcription polymerase chain reaction (qRT-PCR) was performed on a CFX Connect Real-Time System (Bio-Rad Laboratories, Hercules, CA, USA) using TaqMan MicroRNA Reverse Transcription Kit and TaqMan Fast Advanced Master Mix (Applied Biosystems, Foster City, CA, USA). Primers specific for miR-16, miR-20a, miR-21, miR-155, and miR-191 were designed by Applied Biosystems.

**Evaluating miR-20a expression in tissue samples**

Normal pancreas tissues were removed at surgery for pancreatic neuroendocrine neoplasms.
